# Siberian flying squirrels do not anticipate future resource abundance

**DOI:** 10.1186/s12898-016-0107-7

**Published:** 2016-11-14

**Authors:** Vesa Selonen, Ralf Wistbacka

**Affiliations:** 1 Department of Biology, Section of Ecology, University of Turku, 20014 Turku, Finland; 2 Department of Biology, University of Oulu, 90014 Oulu, Finland

**Keywords:** Resource pulse, Masting, Demographic responses

## Abstract

**Background:**

One way to cope with irregularly occurring resources is to adjust reproduction according to the anticipated future resource availability. In support of this hypothesis, few rodent species have been observed to produce, after the first litter born in spring, summer litters in anticipation of autumn’s seed mast. This kind of behaviour could eliminate or decrease the lag in population density normally present in consumer dynamics. We focus on possible anticipation of future food availability in Siberian flying squirrels, *Pteromys volans*. We utilise long-term data set on flying squirrel reproduction spanning over 20 years with individuals living in nest-boxes in two study areas located in western Finland. In winter and early spring, flying squirrels depend on catkin mast of deciduous trees. Thus, the temporal availability of food resource for Siberian flying squirrels is similar to other mast-dependent rodent species in which anticipatory reproduction has been observed.

**Results:**

We show that production of summer litters was not related to food levels in the following autumn and winter. Instead, food levels before reproduction, in the preceding winter and spring, were related to production of summer litters. In addition, the amount of precipitation in the preceding winter was found to be related to the production of summer litters.

**Conclusions:**

Our results support the conclusion that Siberian flying squirrels do not anticipate the mast. Instead, increased reproductive effort in female flying squirrels is an opportunistic event, seized if the resource situation allows.

## Background

One way to cope with irregularity of resource availability is to adjust reproduction according to the anticipated future resource availability [1–4]. This would be particularly useful in resource pulse systems, where resource levels fluctuate remarkably over time [5]. Due to the unpredictable nature of resource pulses animals may be doomed to boom and bust dynamics with dramatic population decline when the resource pulse is over [5]. Anticipation of the resource pulse [1] or anticipation of resource crash [6] could eliminate the lag in population density normally present in consumer dynamics.

A common cause for fluctuation in recourse levels in forest communities is masting by trees, synchronous production of large seed crops, which dramatically affects the whole forest community [5, 7, 8]. For example, densities of seed predators often peak in spring-summer following the resource pulse from the previous autumn [7, 9, 10]. To optimize reproduction with masting events, it is suggested that in European and North American red squirrels [1, 10], chipmunks [11] and fat dormice [12] a mother may increase reproductive output in summers before mast autumns. However, the role of this behaviour in population dynamics of the species remains uncertain [13, 14]. It also remains unclear how general the anticipation behaviour might be for rodents living in forest communities [15].

In this study we test whether Siberian flying squirrels, *Pteromys volans* (hereafter flying squirrels), which depend upon resource pulses of catkins from deciduous trees [16, 17], are able to anticipate current year’s resources in fall by increasing reproductive output in summer. Earlier red squirrel and chipmunk studies have indicated that summer litters are produced in anticipation, whereas spring litters are less affected by future food conditions [10, 13, 14]. Thus, we focus our analysis on production of summer litters in flying squirrels.

We predict that (1) if flying squirrels anticipate the abundance of food resources available to juveniles in the winter of their first year, the production of summer litters is related to resource levels in the following autumn and winter. If flying squirrels do not anticipate the resource availability (2) the production of summer litters is related to the resource abundance in the preceding winter and spring. In addition to the food availability, climate is an important determinant of animal reproduction [18]. Thus, we also analyse whether (3) temperature and precipitation preceding reproduction affects the production of summer litters in flying squirrels.

## Methods

### Study species and its food

**Fig. 1:**
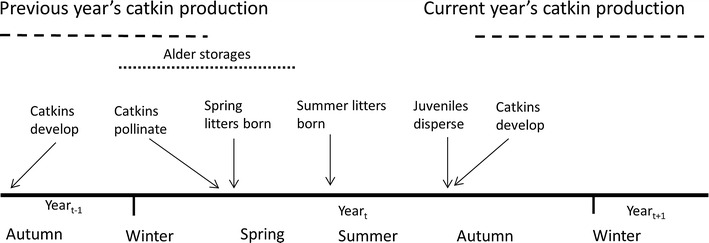
Timeline including the period when alder and birch catkins are consumable (*dashed line*) by flying squirrels, and the timing of birth of flying squirrel spring and summer litters. Only alder catkins are stored, but how long catkins can be stored in caches is not known

**Fig. 2:**
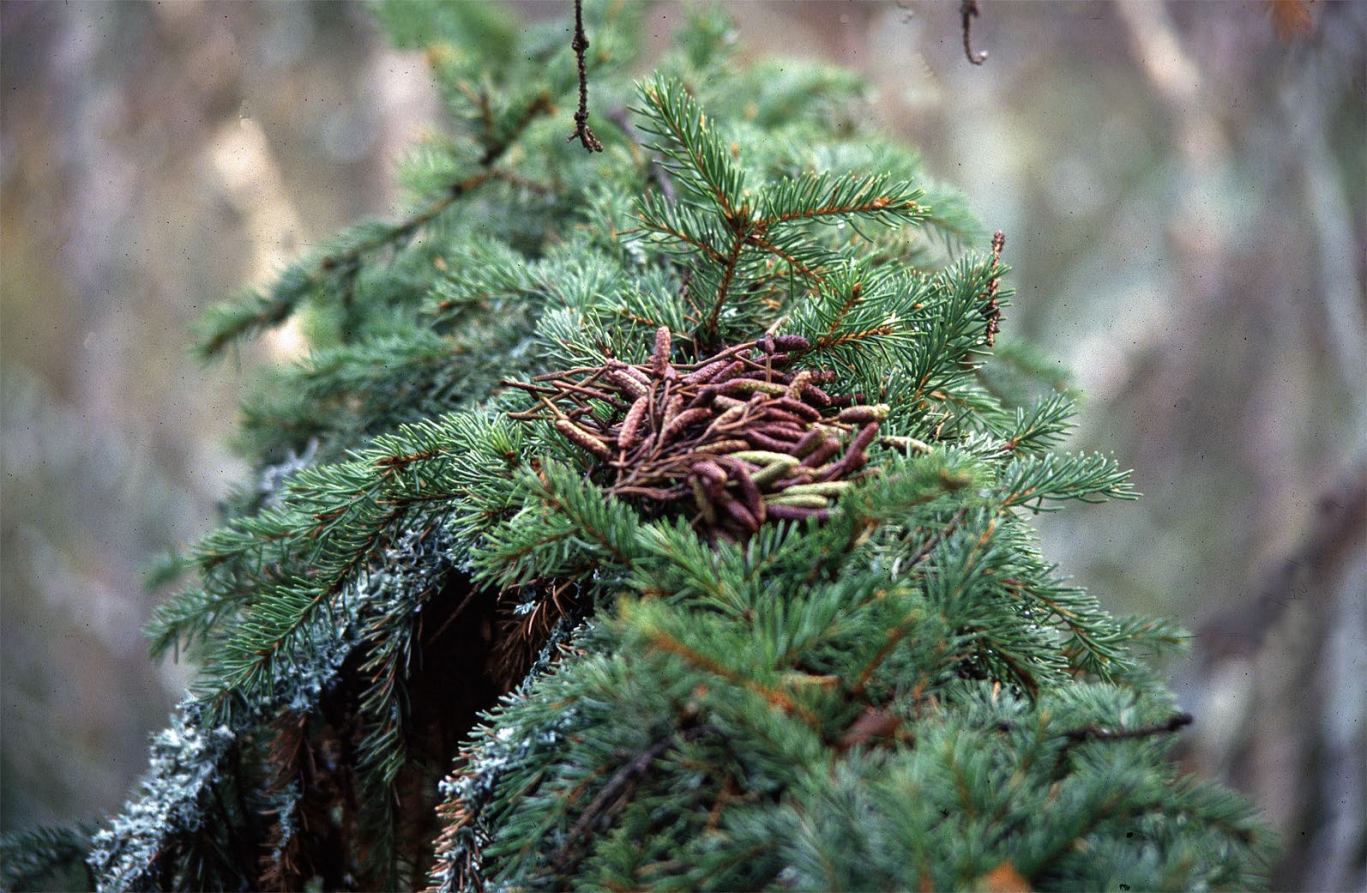
An example of alder catkin storage made by a flying squirrel. Catkins are cached typically on branches of spruces high up in trees, as in this case, and also sometimes in cavities and nest-boxes. An individual can make several different caches. ©Pertti and Risto Sulkava

The Siberian flying squirrel is a nocturnal, arboreal rodent, which nests in tree cavities, nest-boxes and dreys in spruce-dominated boreal forests. The flying squirrel feeds in deciduous trees that occur within spruce forests, birch, *Betula pubescens*/*pendula*, alder, *Alnus incana/glutinosa*, and aspen, *Populus tremula*, being the only used deciduous trees in our study areas [19–21; own observation]. During winter and early spring, when flying squirrel mating, pregnancy and parturition of spring litters occur, birch and alder catkins are the main food of flying squirrels [19, 22; Fig. 1]. Birch catkins form the main part of the winter diet (80% of used food, based on faecal diet analysis; [20]), but only alder catkins are stored and are preferred over birch based on analysis of use versus availability [20–22; Fig. 2]. Catkins begin development during the previous summer, and flying squirrels may start to consume them in autumn [20], continuing to do so during the following winter and early spring (Fig. 1). Catkins flower in spring and birch catkins, which are not stored, are not available when reproductive decisions for summer litters are made. How long alder catkins can be stored in caches is not known, such storage prolongs the time period that catkins are edible, as it prevents catkins from flowering. Catkin production varies considerably between years [16, 17]; see 23] for frequency of pulses in our study areas. Catkin production increases when the previous summer has been warm, however, trees seldom manage to produce mast for two successive years [17, 23]. After the opening of leaves, on average in the beginning of May in our study areas, leaves form major part of late spring and summer diet, together with flower buds of conifers [20, 21]. However, during pregnancy and parturition of spring litters, females may still use catkins, since females are in oestrus and mating occurs starting from mid-March. Spring litters are born in late April. After the spring litter, the second (summer) litter may be born in June, gestation starting in May [24]. Females seem to be territorial, living in separate on average 7 ha home ranges, but males live in overlapping on average 60 ha home ranges encompassing several males and females [25].

### Study areas and data gathering

The studies on flying squirrels were done with individuals living in nest-boxes in two study areas located in western Finland: Luoto (63°49′N, 22°49′E) and Vaasa (63°3′N, 22°41′E), located about 100 km from each other. We do not know any obvious behavioural or reproductive differences between individuals living in nest-boxes, dreys, or natural cavities [26], nor in predator community between sites. The entrance-hole diameter of nest-boxes was 4.5 cm. This entrance-hole size prevents main predators (the pine marten, *Martes martes*, and large owls) entering the nest box. Nest-boxes were made from a piece of aspen or spruce trunk, so that they resembled natural cavities. Natural cavities were rare in our study areas (on average 0.1 cavities per hectare based on 742 spruce forest hectares surveyed within our study areas).

In Luoto flying squirrels were studied during 1993–2014 within an area of 44 km^2^, where between 300 and 400 nest-boxes were placed for flying squirrels. The main forest types in Luoto are shoreline spruce-dominated mixed forests, clear-cuts, and cultivated Scots pine forests. In Vaasa the marking of flying squirrels started in 1992. The Vaasa study area was 25 km^2^ and is covered by spruce forest patches, clear-cuts, and agricultural fields (for more information see [26, 27]. 200–400 nest-boxes were placed within the Vaasa study area to be used by flying squirrels during the study period.

We placed flying squirrel nest-boxes in forest patches of various sizes in sets of 2–4 nest-boxes per site, on average two nest-boxes per mature spruce forest hectare. Box occupancy percentage by the flying squirrel was low (25% nest box occupancy), that is, in most cases a nest box was empty when checked. Flying squirrels were captured by hand in nest-boxes, sexed, weighed, and marked with ear-tags (Hauptner 73850, Hauptner, Germany). The nest-boxes were checked during two sessions in June and August. The latter session was for locating summer litter juveniles. All boxes were checked in spring, but on our second (August) nest box session we focused only on nest box sites occupied by females during the spring (June) nest-box check.

We calculated the number of summer litters occurring in both study areas each year (Table 1), spring litter production of flying squirrels is analysed in [23]. For analysis of summer litters we only included cases where the female was witnessed to successfully produce the spring litter juveniles close to weaning age. In some cases we observed only late born litters without knowledge whether the mother had produced the spring litter. These cases were omitted from the data, because we did not know whether we missed the spring litter (it could be in drey, i.e. a twig nest) or whether the female failed to produce the spring litter. Number of omitted litters was on average 1.1 ± 0.9 litters in Luoto and 1.3 ± 1.5 litters in Vaasa per year. The occurrence of these omitted litters was positively correlated with the number of summer litters born to mothers with observed spring litter each year (estimate 0.15 ± 0.07; F_40_ = 4.80, p = 0.03). During the first nest-box checking session (mean date 14th June) litters had not been weaned (mean weight 59 ± 11 g). The summer litters were on average 56 ± 12 g during the second nest box checking session on average in 18th of August (during this time spring litter juveniles are around 100 g; adult body mass is usually 100–150 g).

**Table 1:** Data for spring litters (n = 640) and summer litters (n = 93) within the two flying squirrel study areas

	Vaasa (mean ± SD)	Luoto (mean ± SD)
Spring litters		
No of litters^a^	n = 404, 18 ± 11 per year	n = 236, 12 ± 6 per year
Litter size^b^	2.5 ± 0.72	2.5 ± 0.86
Body mass^c^	58 ± 11 g	60 ± 10 g
Summer litters		
No of litters	n = 70, 3.3 ± 3.5 per year, min 0, max 12	n = 23, 1.2 ± 1.1 per year, min 0, max 4
Litter size^b^	2.3 ± 0.8	2.6 ± 0.7
Body mass^c^	54 ± 11 g	59 ± 12 g
Years studied	1992–2014	1993–2014^d^

^a^Number of sites with spring litters and checked to locate the possible summer litter

^b^Mothers with summer litter: 2.48 ± 0.65, n = 88 and Mothers without summer litter: 2.52 ± 0.8, n = 547

^c^Body mass at capture on average 14th of June for spring litter and 18th of August for summer litter

^d^Luoto: years 2007 and 2008 omitted due to lack of data

### Food abundance indices

We used estimates from an annual birch-catkin survey conducted by the Finnish Forest Research Institute [28] to estimate food available to flying squirrels each year. These data describe nation-wide pollen availability in Finland. Birch catkins were counted in winter from seed-crop observation stands. The catkin data originated from 15 permanent research stands, where catkins were counted from 30 to 50 birches per stand. Observations were made repeatedly from the same individual trees each year [28]. At our Vaasa study site, a seed-crop observation stand was located within the study area. The closest seed-crop observation stand to our Luoto study site was the Vaasa observation stand located 90 km away. Thus, we used Vaasa indices for both of our study areas, since according to previous analysis of this catkin data, correlation between two sampling sites at this distance is high (r ≈ 0.7), because catkin production of deciduous trees is spatially correlated at scales of up to few hundred kilometers in Finland [16]. Although the food index for Luoto is less accurate than for Vaasa, it describes the yearly variation in catkin production in the area. Both study areas located in coastal area with very similar weather conditions.

For alder there was no catkin count data, but as a proxy we used aerial pollen estimates that correlate with catkin production [16]. Pollen data was collected by the aerobiology unit at University of Turku. Pollen samples were collected from 10 different locations in Finland with EU standard methods and Burkard samplers. The data consisted of accumulated sums of average daily counts of airborne pollen in 1 m^3^ of air during spring (16; http://www.siitepoly.fi/en/). Similarly as above for birch catkin data, we used Vaasa sampling site for both of our study areas. Alder pollen and birch catkin data are correlated, albeit not very strongly (r^2^ = 0.31 for years 1992–2014 in our dataset).

### Weather indices

We used weather information from the closest weather station maintained by the Finnish Meteorological Institute to both study areas. For Vaasa the closest weather station was located within our study area, and for Luoto it was 10 km southeast of the study area. Weather recording stations were at the same altitude with study areas.

We used monthly average weather indices from November prior to gestation to June following lactation. We selected the following periods: For winter weather, we used average temperature and the amount of precipitation in December–January (early winter) and the average temperature and amount of precipitation in February–March (late winter) in our analysis. For spring weather, instead of monthly average temperatures, we used (1) the start date of the growing season, that is, the date after which the average daily temperature in spring was permanently above +5 °C. Additionally, we used (2) growing degree days in April and May (the sum of degrees that in daily average temperature were above 5 °C in a given month). These indices were assumed to describe spring conditions better than mere temperature, although we also tested the effect of temperature in April–May. Temperature permanently above +5 °C is determined to indicate start of growing season by Finnish Meteorological Institute (http://en.ilmatieteenlaitos.fi/seasons-in-finland) and has been observed, for example, to well correspond to birch bud burst in Finland [29]. Lastly, we used precipitation in April–May and temperature and precipitation in June (summer) in our analyses.

### Analysis

Despite the obvious correlations between different weather and resource data, the explanatory variables were relatively independent from each other. We did not allow the variables, past birch and start of growing season, in the same model. This resulted in low collinearity between variables (Variance inflation factor values <2, Proc Reg, SAS 9.3).

To analyse the effects of different food and weather variables on occurrence of summer litters, we used multi-model inference based on Akaike’s information criterion (AIC, smaller values being better). We used AICc values designed for a small sample size and did not include more than three explanatory variables at a time to the model to avoid over-parameterisation. This was done because, in this analysis, the sampling unit was a year. If there was no single clear best fit model or parameter, we used model averaging, using cut-off ΔAIC of 10 and including all models where the term of interest appeared [30]. From the results of model averaging, we considered a parameter to be important in explaining squirrel reproduction if its coefficient and associated 95% confidence interval did not include zero (the obtained results were the same, if we used generalized linear models, analysis not shown). We built models with binomial distribution with GLIMMIX (SAS), using the events/trial option, such that the ‘event’ was the number of summer litters observed and the ‘trial’ was the total number of sites that had a spring litter and that were inspected for a possible summer litter in each study area each year. The explanatory variables were future (current years’ autumn and winter following lactation and weaning) and past (previous winter and spring preceding gestation) catkin production of birch or pollen estimate of alder and aspen (proxy for catkin production) and above described temperature and precipitation estimates before reproduction. The study area was selected as a class variable in the model.

To gain further information on recourse availability/female condition before production of summer litters, we compared body mass of spring litters born to mothers with summer litters and spring litters born to mothers without summer litters. If spring litters were large when observed (born earlier and/or grown faster) that indicates good resource situation before reproduction [23]. For this analysis we only used litters weighed during the same day each year (body mass was calculated as an average for a litter). In addition, we tested whether the age of mother affected its likelihood to have a summer litter. For this analysis we used only females ear-tagged as juveniles, so that the exact age of individual was known. Whether or not a female was observed to produce one or two litters a year was a dependent variable (binomial distribution). The age of the mother as well as the study area were selected as explanatory variables. The ID of the mother was a random variable using Kenward-Roger method to determine degrees of freedom. Finally, with binomial model we tested whether or not a female had summer litter was related to the size of its spring litter. In this model individual ID and year were random variables; study area was included as class variable. The above analyses were done with generalized linear mixed models in GLIMMIX, SAS.

## Results

**Fig. 3:**
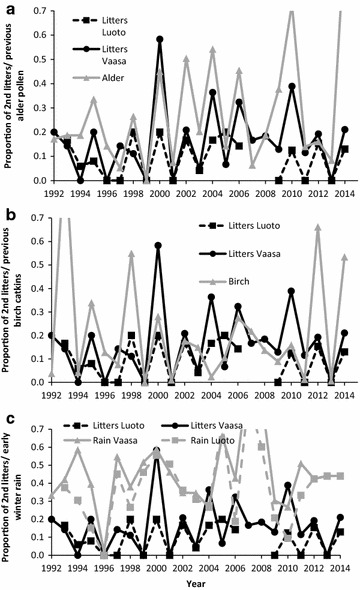
Yearly variation in proportion of observed summer litters of flying squirrels in two study areas in western Finland and **a** alder pollen (proxy for catkin production) and **b** birch catkins in winter/spring preceding reproduction. **c** Rain in early winter preceding reproduction. Alder, birch and rain scaled to values between 0 and 1. Missing data for Luoto for years 2007 and 2008

**Fig. 4:**
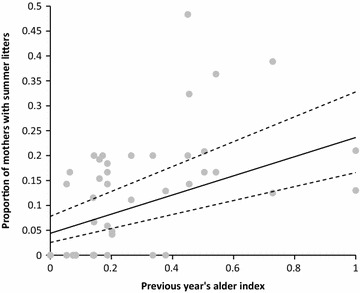
Effect of alder pollen in preceding spring on production of summer litters by flying squirrels. *Solid line* based on predicted values with upper and *lower lines* for confidence interval from best fitted model based on multimodel inference. Alder scaled to values between 0 and 1. *Grey dots* for raw data

We had data for 547 females with only a spring litter and 93 females with both summer and spring litters (total 733 litters; Table 1). Thus, about 15% of mothers were observed to produce summer litters (Figs. 3, 4). Litter sizes were quite similar between summer and spring litters (Table 1) and the size of spring litter was not related to likelihood to produce a summer litter (F_1,323.8_ = 1.3, p = 0.24; Table 1). The mother’s age (age range 1–6 years) was not related to the likelihood of producing summer litters (n = 111 cases; F_1,24_ = 0.32, p = 0.58).

Summer litters were not produced in anticipation of the future resource availability in the autumn and winter of a juvenile’s first year. Instead, alder catkin production during winter and spring before reproduction was significantly related to occurrence of summer litters (coefficient 1.5, 95% CI 1.1 and 2.1; Fig. 4). The top models explaining the occurrence of summer litters included also birch catkins before reproduction and early winter rain (Fig. 3; Table 2). The effect of early winter rain was significant in the model (coefficient 2.1, 95% CI 1.0 and 3.1) but birch had no obvious effect (coefficient 0.29, 95% CI −0.02 and 0.6). Future alder and birch estimates clearly lowered the model fit (Table 2; increase in ΔAIC, alder: 26, birch: 29). Future alder pollen production had a significant, but negative, effect (coefficient −0.30, 95% CI −0.05 and −0.65; Fig. 4), because a mast year with a high number of summer litters is typically followed by a low resource year (Fig. 3). Body mass of juveniles in spring litters born to mothers with summer litters was on average 5 ± 6 g larger than body mass of juveniles in spring litters born to mothers without summer litters (difference to expected 0 g difference: t_45_ = 5.4, p < 0.0001). In other words, if a female produced summer litter, her spring juveniles were born earlier or grew faster compared to juveniles of females who did not produce summer litters.

**Table 2:** Ranking of the best candidate models to explain occurrence of flying squirrel summer litters in Vaasa and Luoto study areas between 1992 and 2014

Model^a^	AICc	ΔAICc	AICc weight
Alder_previous + Rdecjan	117.3	0	0.26
Alder_previous + Rdecjan + birch_previous	117.5	0.8	0.20
Alder_previous + Rdecjan + Tfebmar	119.3	2.0	0.09
Alder_previous + Rdecjan + aspen_previous	119.4	2.1	0.09
Alder_previous + Rdecjan + DdaysApril	119.5	2.2	0.09
Alder_previous + Rdecjan + DdaysMay	119.8	2.5	0.07
Alder_previous + Rdecjan + TJune	119.9	2.6	0.07
Alder_current + Rdecjan	143.5	26.2	0

The best model for both the future and the past food availability are shown. The AICc value, as well as the change in AICc (ΔAICc) and relative weight of support (AIC weight) are shown for each model. Models with cumulative *Wi* = 0.90 presented

^a^Variable names: *T* temperature in given month; *R* rain in given month; *decjan* December–January; *febmar* February–March; *Ddays* degree days; *Aspen* Aspen pollen estimate; *Alder* alder pollen estimate; *Birch* birch catkin estimate; *previous* pollen/catkin estimate available preceding gestation; *current* current years’ pollen/catkin estimate available after lactation and weaning. Study area was included in all models, and it had a significant effect (coefficient and c.l. > 0), since the proportion of summer litters was low in Luoto likely due to a lower density of nest-boxes in Luoto than in Vaasa

## Discussion

We observed that flying squirrels reproductive investment did not anticipate future resource availability. Instead, food levels before reproduction explained increased reproductive effort, in the form of summer litters. In addition, females who produced summer litters had managed to produce spring litters earlier than females who only produced spring litters. This further supports the conclusion that production of summer litters is related to the condition of a female before reproduction.

Our results support the hypothesis that reproductive decisions are determined by the condition of females at the time of reproduction. This kind of behaviour is typical in, for example, income breeding species, like grazers depending on spring plant growth [31] or insectivorous birds [32]. Foraging behaviour of flying squirrels differs from the behaviour of these species since, in winter and spring flying squirrels depend on food that has already developed during the previous autumn, i.e. an example of a capital breeder strategy. It seems likely that storages of alder catkins are important for fuelling reproduction of flying squirrels in summer, since the alder was more clearly related to reproduction than was birch catkin production, an important, but not cached, winter food.

After successfully weaning the spring litter, reproducing again during the same summer seems to require good environmental conditions. The observed relationship between food resources and production of summer litters was clear (Figs. 3a, 4). However, the proportion of mothers with summer litters may be slightly underestimated, since it is possible that we missed a few summer litters, if some females moved from nest boxes to dreys (twig nest). In particular in the Luoto study area, the low number of summer litters is likely due to a lower nest-box density in this study area than in the Vaasa study area [26], which lowers the likelihood of finding summer litters. Nevertheless, both study areas gave similar support for the effect of past alder catkin availability on production of summer litters in flying squirrels.

Weather was also linked to production of summer litters in flying squirrels. Surprisingly, precipitation in winter prior to gestation, not the temperature in spring or summer, was linked to the occurrence of summer litters. It remains unclear what is behind this observed correlation, and in the time-series of the data (Fig. 3c) the relationship was not very clear. However, the lack of sufficient soil moisture is an important stress factor for deciduous trees [33], and it is possible that dry or snowless winter conditions affect moisture conditions and consequently flowering buds or leaves in spring and summer, which provide food for flying squirrels. Indeed, the quality of summer food is a likely candidate that affects summer reproduction of the species. Unfortunately, we were unable to directly study this, but leaf growth is tightly linked to weather conditions during the time period included in our analysis. In any case, the effect of weather on production of summer litters needs further study due to correlative nature of our analysis.

Our results from flying squirrels provide an example of forest-dependent rodent species not able to anticipate a mast. This result is in contrast to observations in some other studies on rodents [1, 10, 11, 34, 35]. For example, North American red squirrels [1] are likely more dependent on cached food than Siberian flying squirrels. North American red squirrels clip new spruce cones containing seeds each autumn and cache them in a larder hoard called a midden [36, 37]. The dependency on middens [38] might increase the adaptive reasons to anticipate the mast in North American red squirrels. However, anticipation is suggested to also occur in forest rodents other than North American red squirrels [10, 11, 34]. The adaptive reasons to anticipate the mast should occur also in flying squirrels as the production of food consumed by flying squirrels is quite similar to that of, for example, Eurasian red squirrels [10]. Flying squirrels start to consume catkins in autumn and continue to do so during the following winter and early spring, when the catkins flower. Thus, if a female could anticipate the coming mast, its offspring would face the winter with optimal resource availability. In addition, variance in birch and alder catkin production [23] is comparable to variation in spruce cone production used by red squirrels [1, 14]. Furthermore, the Siberian flying squirrel are entirely dependent on trees, and very seldom move on the ground (North American flying squirrels, *Glaucomys* spp., move regularly on the ground, e.g. when they harvest truffles). In winter the only foods available for flying squirrels are catkins and buds. However, during summer food other than catkins seems to be sufficient for reproduction as some summer litters were also produced following poor catkin winters. Thus, mast conditions do not appear to be essential for the production of summer flying squirrel litters.

For species observed to anticipate mast, it has previously been speculated that buds that eventually develop into cones/seeds are used to predict the future resource availability [1, 36]. For example, the edible dormouse, *Glis glis*, has been suggested to use the flower buds of the European beech, *Fagus sylvatica*, in spring as a sign of mast [12, 35]. The dormice gain energy from eating these buds, and it has also been observed that food supplementation in spring increases the summer production of this species ([12, 35]; however, for North American red squirrels see [1, 39]). Similarly, in flying squirrels, abundant food resources in the spring were positively correlated with the production of summer litters. However, for dormice the situation is different, as increased energy from flower buds also correlates with a future good seed situation that will benefit the offspring the next autumn and winter [40, 41]. With flying squirrels, the juveniles from summer litters will face the winter without catkins, since mast is generally followed by poor investment in reproduction by trees [42]. This may be problematic, since the survival rate of rodent juveniles is generally highest in mast conditions [43].

## Conclusions

We observed that Siberian flying squirrels do not anticipate the coming mast, but instead adjust their reproductive decision based on current and past food availability. For flying squirrels, an increased reproductive effort is simply a consequence of favourable environmental conditions, which allow females to increase offspring production. The reproductive strategy of Siberian flying squirrels appears to be an opportunistic strategy, depending on the current resource availability, without possibilities to anticipate the future conditions the offspring will face when they mature.
